# Irradiance controls photodynamic efficacy and tissue heating in experimental tumours: implication for interstitial PDT of locally advanced cancer

**DOI:** 10.1038/s41416-018-0210-y

**Published:** 2018-10-24

**Authors:** Gal Shafirstein, David A. Bellnier, Emily Oakley, Sasheen Hamilton, Michael Habitzruther, Lawrence Tworek, Alan Hutson, Joseph A. Spernyak, Sandra Sexton, Leslie Curtin, Steven G. Turowski, Hassan Arshad, Barbara Henderson

**Affiliations:** 1 Photodynamic Therapy Center, Roswell Park Comprehensive Cancer Center (Roswell Park), Buffalo, NY USA; 2 Department of Cell Stress Biology, Roswell Park, Buffalo, NY USA; 3 Department of Biostatistics and Bioinformatics, Roswell Park, Buffalo, NY USA; 4 Translational Imaging Shared Resource, Roswell Park, Buffalo, NY USA; 5 Laboratory Animals Shared Resources, Roswell Park, Buffalo, NY USA; 6 Department of Head and Neck Surgery, Roswell Park, Buffalo, NY USA

**Keywords:** Oncology, Cancer

## Abstract

**Background:**

Currently delivered light dose (J/cm^2^) is the principal parameter guiding interstitial photodynamic therapy (I-PDT) of refractory locally advanced cancer. The aim of this study was to investigate the impact of light dose rate (irradiance, mW/cm^2^) and associated heating on tumour response and cure.

**Methods:**

Finite-element modeling was used to compute intratumoural irradiance and dose to guide Photofrin^®^ I-PDT in locally advanced SCCVII in C3H mice and large VX2 neck tumours in New Zealand White rabbits. Light-induced tissue heating in mice was studied with real-time magnetic resonance thermometry.

**Results:**

In the mouse model, cure rates of 70–90% were obtained with I-PDT using 8.4–245 mW/cm^2^ and ≥45 J/cm^2^ in 100% of the SCCVII tumour. Increasing irradiance was associated with increase in tissue heating. I-PDT with Photofrin^®^ resulted in significantly (*p* < 0.05) higher cure rate compared to light delivery alone at same irradiance and light dose. Local control and/or cures of VX2 were obtained using I-PDT with 16.5–398 mW/cm^2^ and ≥45 J/cm^2^ in 100% of the tumour.

**Conclusion:**

In Photofrin^®^-mediated I-PDT, a selected range of irradiance prompts effective photoreaction with tissue heating in the treatment of locally advanced mouse tumour. These irradiances were translated for effective local control of large VX2 tumours.

## Background

Patients with refractory locally advanced head and neck cancer (HNC) face dire prognoses.^1^ Combination chemotherapy yields objective response rates of 10–36%.^2^ Re-irradiation is associated with significant toxicity.^3–6^ Salvage surgery is associated with prolonged hospitalisation, and the cure rates are poor.^7,8^ The newest immunotherapy regimens result in 5–16% overall response.^9,10^

Interstitial photodynamic therapy (I-PDT) is an alternative treatment for patients with locally advanced cancer that failed to respond or are not amenable to standard of care therapies.^11^ The mechanism of action of PDT involves the generation of reactive oxygen species and radicals through photoactivation of a photosensitiser (PS) in the presence of oxygen.^12^ Intratumoural illumination is accomplished by delivering laser light through optical fibers placed within the target tumour.^11^ In the European Union, I-PDT with temoporfin is approved for palliation in patients with locally advanced HNC that failed to respond to standard therapies.^13–15^ The US Food and Drug Administration (FDA) approved PDT with porfimer sodium (hereinafter referred to as Photofrin^®^) for oncologic use in the treatment of completely obstructing esophageal cancer, partially or completely obstructing non-small-cell endobronchial cancer, as well as high-grade dysplasia associated with Barrett’s esophagus.^12^ The FDA specifies that Photofrin^®^ should be activated with laser light delivered through optical fibers with a cylindrical-diffuser end emitting 630-nm light at an intensity of 400 mW per cm (mW/cm) and energy of 50–300 J/cm per length of the diffuser.^16^

Multiple in vivo studies with several PSs including Photofrin^®^ have shown that light dose rate (i.e., fluence rate, or irradiance, mW/cm^2^) is a major factor in determining the efficiency of the photoreaction via its effect on oxygen consumption.^17–20^ In those studies, the light was delivered to the tumour surface with external beam PDT (EB-PDT), where it was demonstrated that irradiance >150 mW/cm^2^ could significantly deplete tumour oxygen levels and result in a ineffective tumour destruction.^21^ For I-PDT of the prostate, multiple investigators have suggested that intratumoural light dose (i.e., light fluence, J/cm^2^) is the key parameter to achieve an effective tumour control.^22–25^ To the best of our knowledge, the impact of intratumoural light irradiance (thereafter referred to as irradiance) on response and local control has not been reported for Photofrin^®^-mediated I-PDT.

An early I-PDT study in mice drew attention to the fact that very high irradiances can induce thermally ablative temperatures (>50 °C).^26^ Subsequently, the emphasis of researchers and clinicians was to use irradiance ≤150 mW/cm^2^, which were believed to minimise tissue heating.^12,27^ However, the current clinically prescribed light intensity for I-PDT with Photofrin^®^ is 400 mW/cm, which can be expected to result in an irradiance that will induce considerable tissue heating.

In order to improve and optimise I-PDT, it is imperative to define the relationship between tumour response and intratumoural irradiance and fluence, and to understand the extent and contribution of light-induced tissue heating (LITH), including ablative temperatures, to treatment success or failure; an understanding that is thus far lacking. We report here the results from a preclinical study that elucidates and quantifies the impact of intratumoural irradiance on tumour response and cure, toxicity, and photothermal effects in I-PDT with Photofrin^®^.

## Materials and methods

### Animal and tumour models

All procedures were carried out in accordance with a protocol approved by the Institutional Animal Care and Use Committee at Roswell Park Comprehensive Cancer Center.

#### Mice bearing syngeneic locally advanced squamous cell carcinomas (SCCVII)

The SCCVII is a poorly immunogenic, syngeneic, murine tumour model that is appropriate for studying treatments for HNC.^28,29^ Female C3H mice, 8–12 weeks old, were obtained from the Charles River Laboratories, Frederick, MD. Animals were housed in micro isolator cages within the Cancer Center’s animal facility and provided food and water ad libitum. A suspension of 10^6^ SCCVII cells was prepared (100 µL injection volume) and injected subcutaneously into the shoulder region of the mice. Mice were treated when SCCVII tumours reached a volume of 400–600 mm^3^, a size of a locally advanced murine tumour.^30^

Tumours were measured three to four times weekly, and cure was defined as complete regression of the tumour for at least 60 days. Animals were killed at 60 days after treatment or when the tumour volume reached ≥4000 mm^3^. In this tumour model, all local recurrences after treatment were palpable within 30 days.

#### VX2 squamous cell carcinomas in New Zealand White rabbits

This is a well-accepted preclinical model to practice clinically relevant minimally invasive ablation techniques of cancerous tumours.^31–33^ A piece of VX2 tumour from a donor rabbit, of about 1 mm^3^, was surgically implanted into the sternomastoid muscle at the neck level of each New Zealand White (NZW) rabbit (specific pathogen-free NZW rabbit, Charles River Laboratories). Non-contrast-enhanced computed tomography (CT) scans were performed to assess tumour volume and determine the presence or absence of metastases. I-PDT was scheduled when tumours reached a size of 2–3 cm in their longest dimensions. Following treatment, animal health and the treatment site were monitored daily. Tumour response was determined with CT every 1–2 weeks.

#### Determination of Photofrin^®^ concentration in SCCVII and VX2 tumours

We determined the Photofrin^®^ concentrations in excised tumour tissue by fluorescence measurements following tissue solubilisation, as previously described.^34^ Tumour tissue samples were collected 24 h post injection of Photofrin^®^ in animals bearing a single tumour similar in size and location to the one treated in the study cohorts. This assay provides an average concentration of the Photofrin^®^ in the tumour.

#### Evaluation of necrosis and hypoxia in SCCVII tumours

Standard hematoxylin and eosin (H&E) staining was used for examining tumour tissue morphology. The Hypoxyprobe™-1 Plus Kit containing pimonidazole hydrochloride (Hypoxyprobe, Inc., Burlington, MA) was used to examine tumour hypoxia, following the Kit instructions. The animals were killed via cervical dislocation and tumours were excised immediately to minimise hypoxia. Positive controls (for hypoxia staining) were prepared following Busch et al.^19^ Three stained sections from each tumour were examined for morphology and pimonidazole binding at ×40 magnification with an optical microscope (Axiovert 200, Carl Zeiss Inc., Thornwood, NY).

#### Computer simulations of light propagation in tissue

Our finite-element modeling (FEM) approach was employed to compute intratumoural light irradiance and fluence, as previously described.^35^ The model was validated by comparing the simulation results to measurements in liquid phantoms with optical properties similar to mouse tumour.^35^ The simulations were done for geometrical models of tumours with cylindrical diffuser and catheters. The models were reconstructed from representative magnetic resonance imaging (MRI) scans of mouse tumours and CT scans of NZW rabbit with VX2 tumour. The scans were segmented with image analysis software (Simpleware, Exeter, UK), as previously described.^35,36^ The tumour optical properties were measured with a non-invasive reflectance spectroscopy system (Zenascope™, Zenalux Biomedical, Durham, NC). The FEM simulations were conducted by solving the light diffusion equation in 3D with Comsol 5.2 (Comsol, Inc., Burlington, MA). The governing equations and model assumptions are detailed in our previous publications.^35,36^

#### Interstitial light and photodynamic therapy

The laser light was delivered through 0.98 mm diameter optical fibers with a 2-cm long cylindrical diffuser (RD20, Medlight SA, Ecublens, Switzerland) connected to 1.0 W diode-laser module that emits 630 ± 3 nm light (ML6500–630, Modulight, Inc., Tampere, Finland). The laser power, emitted from the cylindrical diffuser, was measured with an integrated sphere (Integra, Coherent, Auburn, CA) before and after treatment.

C3H mice with SCCVII tumours were treated under isoflurane (3–5%) anesthesia. In one set of studies, mice were treated with a light energy of 100 J/cm that was administered with light intensities of 60, 100, 150, 200, or 400 mW/cm, with and without 5 mg/kg Photofrin^®^. The laser light was delivered through a single RD20 that was placed in a light transparent catheter (18G shielded IV catheter; Becton, Dickinson and Company, Franklin Lakes, NJ). The catheter was previously inserted through the centerline of the tumour along its longest axis and horizontal to the mouse skin.

In subsequent studies, tumour-bearing mice were treated using two cylindrical diffusers placed within two parallel catheters that were inserted at 6 ± 1 mm apart, along the tumour centerline and horizontal to the mouse skin. In this regimen, the light fluence and irradiance were selected in order to test the hypothesis that, when accounting for light attenuation in the tumour, an effective I-PDT requires intratumoural light irradiance and fluence that are comparable to those resulting from effective EB-PDT.^17,37^ The FEM simulations were utilised to identify light intensities for the mouse studies. The simulations suggest that two 2-cm cylindrical diffusers delivering 630-nm light at 60–150 mW/cm and 540 J/cm per fiber are required to deliver hypothetically effective intratumoural irradiance and fluence for I-PDT with Photofrin^®^. Mice were treated with either no drug or with Photofrin^®^ at 3.3, 5.0, or 6.6 mg/kg that was administered via intravenous tail vein injection, 24 h prior to the light delivery. Compared to the clinical use of Photofrin^®^, the higher Photofrin^®^ dose and shorter drug-light interval 24 vs. 48 h, was used to compensate for the higher metabolism of these animals in comparison to humans. A control cohort included mice (*n* = 21) with untreated SCCVII tumours.

In the NZW rabbit study, image-based pretreatment planning with our FEM simulations was conducted for each animal. The images were acquired 1–2 days prior to treatment with non-contrast-enhanced CT (LightSpeed VCT, GE Healthcare). A single bolus of 5 mg/kg Photofrin^®^ was administered intravenously via a catheter in the ear vein 24 h prior to light illumination. The rabbits were treated under general anesthesia with isoflurane inhalation. Surface fiducial markers (IZI Medical Products, Owings Mills, Maryland) were used for guiding the placement of the cylindrical diffuser according to the treatment plan, as detailed in Oakley et al.^36^ A light dosimetry system was used to measure irradiance and fluence during treatment.^35,38^ The tumour temperature was measured during treatments with optical fiber thermometers (FOTemp, Micronor, Inc., Camarillo, CA).

#### Magnetic resonance imaging and thermometry

The temperature changes during treatment of mice were assessed with magnetic resonance thermometry (MRT). The MRT was performed using the proton resonance frequency method, in a 4.7 Tesla preclinical scanner using the ParaVision 3.0.2 MRI (Bruker Biospin, Billerica, MA) with a 35 mm quadrature transceiver coil. A nuclear magnetic resonance tube filled with cottonseed oil was used as a phase normalisation reference. The MRT was performed using a 15-slice spoiled gradient echo scan (echo time/repetition time = 2.85/165 ms, flip angle = 20, averages = 2). Following three baseline acquisitions scans, the interstitial light was administered, and MRT data were collected throughout the treatment and for 5 min after the laser light was off. Raw *k*-space data were filtered with a two-dimensional (2D) Gaussian function to improve signal-to-noise ratio. Following 2D Fourier transforms into the spatial domain, phase images were normalised to the phantom and a 2D phase unwrapping function was applied. The temperature changes were calculated by conversion of 0.0359 radians decrease in phase values for 1 °C increase in temperature. Laser-induced irreversible thermal damage (herein defined as photothermal ablation) will occur immediately when the tissue temperature exceeds 60 °C.^39^ Using the MRT data, we computed the volume fractions that were subjected to temperatures sufficient to induce photothermal ablation during treatment. A computer code written in MATLAB (Mathworks, Inc., Natick, MA) was used to calculate the number of voxels at *T* > 60 °C divided by the number of voxels comprising the tumour.

#### Statistical methods and analysis

The primary end points were 60-day cure or time to the tumour reaching a volume ≥4000 mm^3^ or death. These end points were compared using either exact Pearson *χ*^2^ or Cox regression analysis. The exact Kruskal–Wallis rank sum test was used to compare intratumoural Photofrin^®^ retention for different systemic doses. The percent of tumour volume at *T* > 60 °C was estimated assuming a nonlinear monotone-increasing exponential model and fit using a Newton-Raphson optimisation technique.^40^ A two-way analysis of variance was then used to compare between light-only and I-PDT with Photofrin^®^. All experimental conditions had sample sizes ranging from 10 to 20 animals. All tests were tested at *α* = 0.05 (two-sided). The general testing strategy was to first carry out the overall test at alpha, and if significant, carry out Bonferroni-adjusted pairwise comparisons. All analyses were done using SAS 9.4 (SAS Institute, Inc., Cary, NC, USA). As a guide, at 80% power and *n* = 10 per group in a three group comparison, we could detect cure rates with roughly a 60% or larger difference between groups and hazard ratios (HRs) of roughly 4 to 1 or larger (0.25 or smaller) for our time-to-event end points.

## Results

### Locally advanced SCCVII tumours demonstrate aggressive growth, are minimally hypoxic, and retain Photofrin^®^

An inoculation dose of 10^6^ SCCVII cells produced tumours in 100% of mice. All untreated control tumours reached a volume of 4000 mm^3^ within 10–11 days of tumour cell inoculation; additionally, no palpable tumours showed spontaneous regression. Large murine tumours tend to form central necrosis and regions of tissue hypoxia, which negatively affect the response to PDT.^21,41^ Therefore, we evaluated SCCVII tumour pathology with H&E and pimonidazole staining. Histology and immunohistochemistry revealed little necrotic tissue, with minimally hypoxic regions (data not shown). The intratumoural Photofrin^®^ concentrations in freshly excised SCCVII tumours were 2.7 ± 1.1, 3.8 ± 1.5, and 6.0 ± 1.7 mg/kg at 24 h, corresponding to the time at which I-PDT light is delivered, following intravenous administration of 3.3, 5.0, and 6.6 mg/kg, respectively.

### Interstitial delivery of 630-nm light induces significant tissue heating

**Fig. 1:**
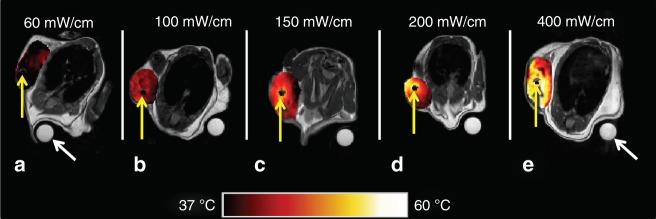
Pseudo-colourised temperature maps (°C) within the tumour as measured by MR thermometry at the end of the laser irradiation for different light intensities. A light energy of 100 J/cm was delivered through a single 2-cm cylindrical-diffuser fiber. There was no difference in the temperature field in mice treated with or without Photofrin. Hence, the images are shown as function of light intensity only (**a**–**e**): 60, 100, 150, 200, and 400 mW/cm. Yellow arrows denote location of treatment fibers. A nuclear magnetic resonance tube filled with cottonseed oil was used as a phase normalisation reference (white arrows). Noteworthy, the non-concentric isotherms, around the cylindrical-diffuser fibers, suggest that the fibers itself was not heated. The increase in temperature was attributed to light absorption by blood, as detailed in the discussion

The impact of light intensity on tumour heating was investigated in mice treated with or without Photofrin^®^. The FDA-approved light intensity of 400 mW/cm was de-escalated to 200, 150, 100, and 60 mW/cm. The corresponding intratumoural irradiances were: 9.1–979, 4.5–490, 3.4–367, 2.3–245, and 1.4–147 mW/cm^2^. The computed minima and maxima were at the margins and treatment fiber/tumour interface, respectively. The MRT data revealed that tumour tissue heating occurred when 100 J/cm of 630-nm light was delivered through single 2-cm cylindrical diffuser at intensities of 60–400 mW/cm (Fig. 1a–e). The non-concentric isotherms around the cylindrical-diffuser fibers suggest that the fibers were not heated.

Having established that tissue heating is an integral feature of I-PDT, we proceeded to analyse its extent and consequences. The light-induced tissue heating (without Photofrin^®^) will be referred to as LITH.

**Fig. 2:**
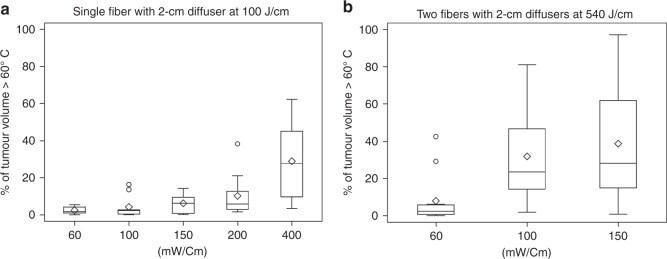
The distributions of percentage of tumour volume >60 °C were summarised using boxplots across the mW/cm settings. **a** Single fiber with 2-cm cylindrical diffuser at 100 J/cm. **b** Two fibers with 2-cm cylindrical diffusers at 540 J/cm. The top, middle, and bottom lines of a box in the boxplots indicate the 25th percentile, median, and 75th percentile, respectively. The “whiskers” in the boxplots represent the range of the data. Open circles in the plot show the values that are beyond 1.5 box lengths from the end of the box

At each of the above specified light intensities, the temperature change and extent of the tumour volume at *T* > 60 °C (photothermal ablation temperature^39^) was the same (data not shown) in mice treated with either LITH or I-PDT with 5 mg/kg Photofrin^®^. Therefore, the analysis of relative tumour volume at *T* > 60 °C, following delivery of 100 J/cm 630-nm light, was combined for LITH and I-PDT. In mice treated with either 60 or 100 mW/cm, <5% of the tumour volume was subjected to *T* > 60 °C (Fig. 2a). At light intensities of 150 and 200 mW/cm, ~15 and 20% of tumours’ volume were at *T* > 60 °C, respectively. At 400 mW/cm, about 10–60% of the tumours’ volume were at *T* > 60 °C. These data suggest that I-PDT can lead to tumour temperatures capable of instantaneous photothermal ablation.

To address the question of whether photothermal ablation or PDT governed tumour response in I-PDT, we compared tumour responses in mice treated with LITH to those treated at the same light intensity with I-PDT using 5 mg/kg Photofrin^®^. We evaluated tumour response in 10 cohorts of mice, at 100 J/cm and 60–400 mW/cm delivered through a single 2-cm cylindrical diffuser. We compared the probability of time-to-event (i.e., tumour volume ≥4000 mm^3^) following I-PDT vs. LITH, using the HR analysis, as shown in Table 1. In this analysis, an HR = 1 means equal tumour growth delay in animals treated with I-PDT or LITH; an HR <1, I-PDT has better outcomes than LITH alone; and for an HR >1, LITH alone has better outcomes than I-PDT. At 400 mW/cm (9.1–979 mW/cm^2^), an HR = 1.57 suggested that LITH governed tumour growth delay, and appears to have negated a possible PDT contribution. At light intensities of 200, 100, and 60 mW/cm, I-PDT appears to be somewhat, although not significantly, more effective than LITH. Only a light intensity of 150 mW/cm (3.4–367 mW/cm^2^) revealed a significant PDT contribution (HR = 0.04).

**Table 1:** The hazard ratios comparing probabilities for time-to-event for interstitial thermal PDT (I-PDT) vs. light-induced tissue heating (LITH); i.e., P(I-PDT)/P(LITH)

Light intensity (mW/cm)^a^	Hazard ratio	Confidence interval^b^
400	1.57	0.15, 15.89
200	0.17	0.015, 1.97
150	0.04^c^	0.003, 0.51
100	0.15	0.01, 1.64
60	0.21	0.02, 2.18

The time-to-event was tumour volume ≥4000 mm^3^ (local tumour control) A hazard ratio = 1 means equal efficacy of the I-PDT and LITH treatments. For a hazard ratio <1, I-PDT was better than LITH for local tumour control; for a hazard ratio >1, LITH provided better local tumour control than I-PDT ^a^Light was delivered with a single 2-cm cylindrical-diffuser fiber ^b^Bonferroni-adjusted confidence intervals accounting for multiple comparisons at alpha = 0.05 ^c^HR was statistically different from 1

### I-PDT with two-fiber illumination increased intratumoural irradiance that significantly improved cure rates in locally advanced SCCVII

**Fig. 3:**
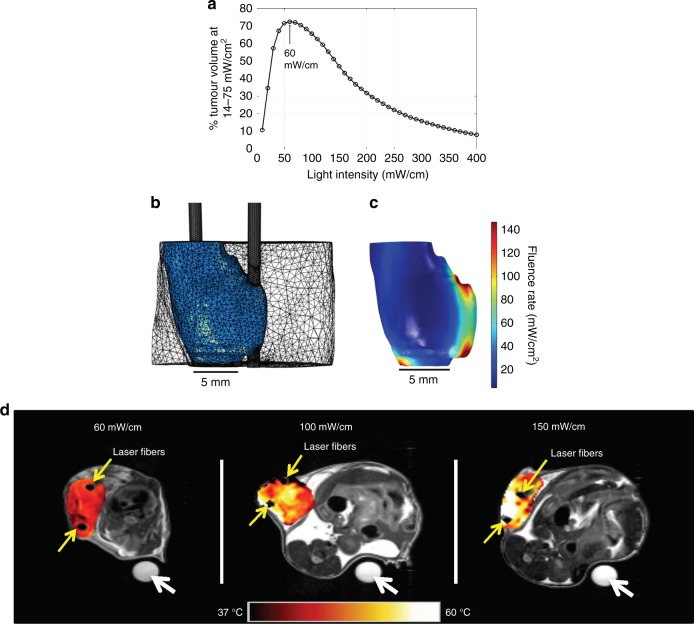
**a** The percent tumour volume that will be illuminated at 14–75 mW/cm^2^ vs. light intensity delivered through two 2-cm cylindrical diffusers at 6.31 ± 0.11 mm apart. **b** The representative geometry (used in **a**) constructed from magnetic resonance imaging of a tumour (556 mm^3^) with two fibers, and the mesh generated in the finite-element modeling. **c** The three-dimensional distribution of the light irradiance for an input light intensity of 60 mW/cm per fiber. The minimum and maximum irradiances were 5.0 and 147 mW/cm², respectively. **d** Pseudo-colourised temperature maps (°C) within the tumour as measured by MR thermometry at the end of the laser irradiation for different light intensities. A light energy of 540 J/cm was delivered through two 2-cm cylindrical-diffuser fibers at 6 ± 1 mm apart. Yellow arrows denote location of treatment fibers. A nuclear magnetic resonance tube filled with cottonseed oil was used as a phase normalisation reference (white arrows)

Turning the focus toward the PDT component in I-PDT, intratumoural irradiance is recognised as a major determinant of PDT effectiveness. Several studies suggest that in EB-PDT irradiance in the range of 14–75 mW/cm^2^ and fluence of 128 J/cm^2^ would result in an effective tumour response in mouse models.^17,37^ Using the results from those studies as a guideline, we calculated the percent tumour volume that would be illuminated at 14–75 mW/cm^2^ vs. light intensity in a representative geometry constructed from an MR image of an average SCCVII tumour (556 mm^3^); calculations were based on delivering I-PDT with two 2-cm long cylindrical-diffuser fibers spaced 6.31 ± 0.11 mm apart. The FEM was carried out using measured absorption and reduced scattering coefficients of 145 1/m and 760 1/m, respectively, which were in agreement with published data for murine tumours.^42^ The index of refraction was set to 1.37, and laser light was 630 nm. The 60 mW/cm appeared to be optimal when the objective was to maximise the PDT contribution by delivering 14–75 mW/cm^2^ to the largest portion (72%) of the tumour volume (Fig. 3a). The intratumoural irradiance distribution (5.0–147 mW/cm^2^) for 60 mW/cm is shown in Fig. 3b, c. At light intensities <60 mW/cm, a larger part of the tumour volume will receive <14 mW/cm^2^, and at light intensities >60 mW/cm, a larger part of the tumour volume will receive >75 mW/cm^2^.

Assuming that in I-PDT the intratumoural fluence should be comparable to the one delivered to the tumour surface in EB-PDT (128 J/cm^2^, over about 9000 s), the light energy should be 540 J/cm at 60 mW/cm. In addition to 60 mW/cm, we evaluated tumour response to 100 and 150 mW/cm with 540 J/cm, which also has the advantage of shorter treatment times.

MRT was used to quantify the intratumoural heating in 60, 100, and 150 mW/cm with 540 J/cm and two 2-cm cylindrical diffusers in six additional cohorts of mice. A substantial LITH was observed within the treated tumour (Fig. 3d). Because the extent of photothermal ablation was the same for LITH and I-PDT, our analyses was for the impact of light intensity only on tumour temperature (*n* = 10 at each setting). There was a significant difference in the volume fraction of tumour >60 °C in mice treated with 5.0–147 mW/cm^2^ (i.e., 60 mW/cm) in comparison to mice treated with 8.4–245 mW/cm^2^ (*p* = 0.0020) or 12.6–367 mW/cm^2^ (*p* = 0.0007) corresponding to 100 and 150 mW/cm, respectively (Fig. 2b).

**Fig. 4:**
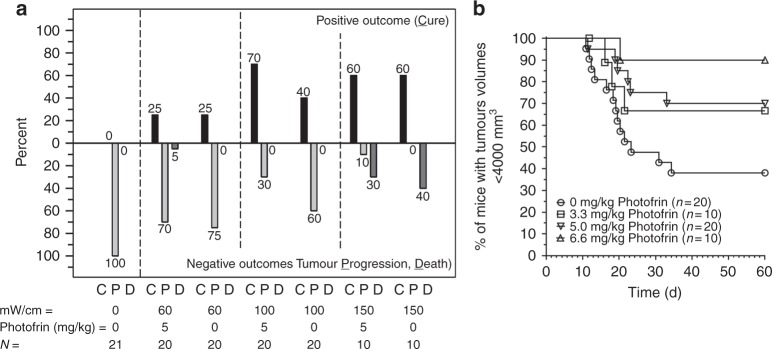
The cure rate (C), tumour progression (P), and death (D) of mice with locally advanced SCCVII treated with interstitial light (630 ± 3 nm; 540 J/cm) delivered through two-optical fibers with a 2-cm cylindrical light diffuser end, placed 6 ± 1 mm apart. **a** Mice treated with light-only or I-PDT with 5 mg/kg Photofrin^®^. **b** Kaplan–Meier plots comparing LITH with I-PDT at 100 mW/cm, 540 J/cm with 3.3, 5.0, and 6.6 mg/kg Photofrin^®^. The I-PDT with 6.6 mg/kg result in a 90% cure that was significantly better (*p* = 0.025) than light-only

The time-to-event mouse studies showed that 25% cure could be obtained with 60 mW/cm and 540 J/cm using two 2-cm cylindrical diffusers (Fig. 4a), and that there was no difference between LITH and I-PDT. However, by increasing the I-PDT light intensity from 60 to 100 mW/cm, 540 J/cm, and 5 mg/kg Photofrin^®^, a significantly (*p* = 0.0104) higher cure rate of 70% was obtained. A 40% cure rate with LITH was observed in mice treated with 100 mW/cm and 540 J/cm (middle panel in Fig. 4a). Increasing the systemic Photofrin^®^ dose to 6.6 mg/kg significantly (*p* = 0.025) enhanced the cure rate to 90% following I-PDT in comparison to LITH, as shown in Kaplan–Meier plots (Fig. 4b). A cure rate of 60% was observed for LITH and I-PDT at 150 mW/cm and 5 mg/kg Photofrin^®^ (right panel in Fig. 4a). However, light intensity was also associated with 30–40% treatment-related death. The death events occurred during or within 24–48 h after treatment. Although not statistically significant, we observed an increase in core temperature (to 41–42 °C) in the few mice that died during treatment.

### FEM translates the mouse study I-PDT data to achieve local control in NZW rabbit with locally advanced VX2 neck tumours

**Fig. 5:**
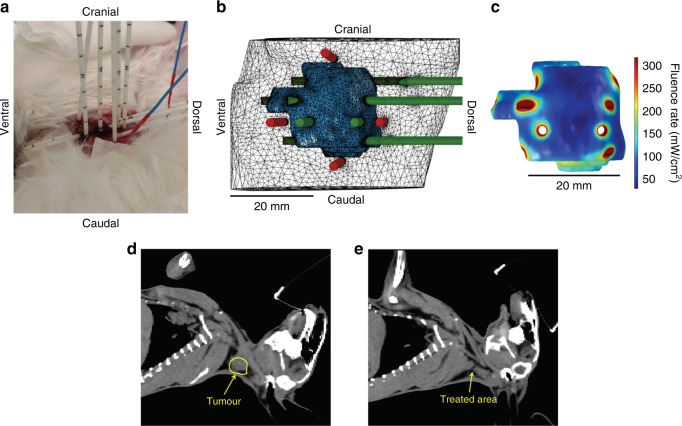
**a** Image of the rabbit treated with I-PDT. Visible in the image are the transparent close-end sharp catheters where treatment or dosimetry fibers were inserted. The blue probes are the optical thermometers that were placed on the tumour surface at margins. **b** 3D representation of the treatment plan. The tumour is represented in blue. The intended treatment fibers are in green and the intended location of dosimetry fibers are in red. **c** 3D representation of the intratumoural irradiance throughout the tumour. **d** CT scan prior to treatment, and (**e**) a CT scan, 13 weeks post treatment, showing no evidence of cancer

To expand our knowledge for I-PDT for tumours with comparable size and location to clinical settings, we used the FEM simulations to guide this treatment in locally advanced VX2 carcinoma. In the mouse study, the high 70% cure rate was obtained with I-PDT (5 mg/kg Photofrin^®^) with two 2-cylindrical diffusers and 100 mW/cm and 540 J/cm, which corresponded to an intratumoural irradiance of 8.4–245 mW/cm^2^ and a fluence of 45–1323 J/cm^2^. The Photofrin^®^ retention in VX2 tumours was 4.0 ± 0.67 mg/kg 24 h after intravenous administration of 5.0 mg/kg Photofrin^®^, similar retention observed in SCCVII in mice. I-PDT with 5.0 mg/kg Photofrin^®^ was used to treat five NZW rabbits with VX2. Because the average VX2 tumour volume was >8–10 times larger than the mouse tumours and the maximum number of fibers we could implant was limited by the anatomy, relatively high light intensities (200–250 mW/cm) were required to deliver ≥8.4 mW/cm^2^. The average minimum of intratumoural irradiance, 20.5 ± 9.1 mW/cm^2^, was higher than in mice. A representative example of I-PDT that resulted in an effective response is shown in Fig. 5. The tumour volume (5200 mm^3^) was treated with 10 optically transparent closed-end brachytherapy catheters (Flexi Needle 14 G, Best Medical International, Inc., Springfield, VA), through which the cylindrical diffuser and/or dosimetry fibers were inserted (Fig. 5a). The tumour geometry with fibers in the computer model, and the FEM-computed intratumoural irradiance are shown in Fig. 5b, c. The FEM was used to guide the placement of the cylindrical diffusers for treatment, as previously described.^35,36^ The minimum irradiance was 30.5 mW/cm^2^. Treatment times were adjusted so that ≥45 J/cm^2^ was delivered to tumour and surrounding margins. The measured maximum temperature was within 34.2–49.0 °C at the tumour surface, indicating intratumoural tissue heating. The rabbit core body temperature did not change during treatment. There were no adverse events during and following treatment. The animal recovered well from the anesthesia and treatment, had normal food intake, and showed no sign of distress or pain. Complete tumour regression was observed within 2–3 days, and no evidence of tumour was seen 13 weeks post I-PDT (Fig. 5d, e). The minimum irradiances and associated tumour responses for all five rabbits are presented in Table 2. This technique was successfully employed in the treatment of the four more NZW rabbits with VX2 tumours in the neck. An effective local control in 4 out 5 VX2 was accomplished with I-PDT delivering ≥16.5 mW/cm^2^ and ≥45 J/cm^2^ to 100% of the tumour volume. In one animal that had regional recurrence, the minimum irradiance was 6.9 mW/cm^2^, well below our criteria for effective irradiance. Light-alone treatments in three additional rabbits resulted in only minimal visible response. More in-depth rabbit studies are still ongoing.

**Table 2:** The minimum irradiances and associated tumour responses observed in I-PDT with intravenous administration of 5 mg/kg Photofrin^®^, 24 h prior to light delivery at 200, 225, and 250 mW/cm through cylindrical-diffuser fibers inserted in large VX2 tumour in the neck of New Zealand White rabbits, as the example in Fig. 5a for RB2017-24

Rabbit #	Minimum intratumoural irradiances in 100% of the tumour volume^a^	Response
RB2017-23	^b^6.9 mW/cm^2^	Local response, with regional metastasis to the salivary gland, and evidence of lung metastasis
RB2017-24	30.5 mW/cm^2^	No evidence of tumour at 13 weeks post therapy. Cure
RB2017-25	^b^25.8 mW/cm^2^	No evidence of tumour at 13 weeks post therapy. Cure
RB2017-30	^b^16.5 mW/cm^2^	Complete local control, evidence of lung metastasis
RB2017-31	^b^22.9 mW/cm^2^	Complete local control, evidence of lung metastasis

The treatment times were adjusted to deliver ≥45 J/cm^2^ in 100% of the tumour volume ^a^The maximum intratumoural irradiance was 318 mW/cm² for RB2017-24, 25, 30, and 31 and was 398 mW/cm² for RB2017-23 ^b^Two sessions of tumour illuminations were conducted sequentially

## Discussion

I-PDT represents a promising strategy for treating large, locally advanced cancers that have failed to respond to conventional therapies.^11^ Here, we describe the results of experimental laboratory animal studies aimed at delineating the relationship between tumour response and intratumoural light irradiance in I-PDT. Our two major findings are:

1. LITH is an inherent component of I-PDT with Photofrin^®^ at irradiance required for local tumour control.
2. We have identified a set of light dosing parameters, which we predict can be translated to the clinical setting to achieve meaningful local tumour control with I-PDT. These parameters are a minimum irradiance of 8.4–16.5 mW/cm^2^ and a fluence of ≥45 J/cm^2^ delivered to 100% of the tumour and margins. It is also recommended not to exceed intratumoural irradiance of 398 mW/cm^2^.

Limiting high-temperature heating effects during treatment has been an objective of PDT.^12^ In EB-PDT, tissue heating is minimised to 2–3 °C with irradiance ≤150 mW/cm^2^.^27^ However, moderate to significant heating is unavoidable in the I-PDT approach that is required to treat bulky disease. The mouse tumour response data, for I-PDT using the FDA recommended light intensity of 400 mW/cm, suggest that photothermal ablation dominated the tumour response. This is because a significant part of the tumour was subjected to *T* > 60 °C (Figs. 1e, 2a), a temperature capable of inducing immediate irreversible thermal damage.^39^ The PDT contribution was lessened, most likely due to the exceedingly high irradiance, up to 979 mW/cm^2^ at 400 mw/cm, that can induce photochemical oxygen depletion and thus impede PDT.^21,41^ LITH appears to be independent from the PDT effect, as the addition of the PS had no impact on the temperature increase in this mouse model. The non-concentric isotherms around the cylindrical-diffuser fibers (Fig. 1a–e) suggest that the cylindrical-diffuser fiber itself does not heat up at light intensities between 60 and 400 mW/cm. The tumour temperature increase was directly proportional to the light intensity, and hence to intratumoural irradiance. Numerous studies have shown that laser light in the red region of the spectrum, including 630-nm light, is converted to heat primarily via absorption by blood constituents.^43^ Our findings suggest that the photothermal ablation in our in vivo models was due to light absorption by endogenous tissue components, possibly oxy-deoxyhemoglobin which, at 630 nm, has a relatively high-absorption coefficient in comparison to other tissue elements.^43^ Yu et al.^23^ showed that a reduction in blood flow will decrease PDT efficacy. Ritz et al.^44^ reported that tissue coagulation will decrease light penetration. These effects, as well as photochemical oxygen depletion, can explain the deficiency of a PDT contribution at very high light intensities and intratumoural irradiance.

It should be noted that in addition to the ablative effects of LITH, our MRT data indicate that hyperthermic temperatures are generated in certain tumour regions and that this may impact the PDT response.^45^ These aspects were not addressed in this study, but are the subject of ongoing investigations.

Although less effective than I-PDT, LITH was able to induce cures in the murine model in the two-fiber regimen (Fig. 4). Instantaneous thermal coagulation occurs when tissue is subjected to *T* > 60 °C,^39^ but the entire tumour was not at that critical temperature (Fig. 2b). The cures were obtained because other parts of the tumours were subjected to 45–60 °C temperatures that will induce cell death over the time (e.g., 2 min at 50 °C), according to the classical thermal dose model.^46^ In the two-fiber regimen, a significant increase in tumour cures in I-PDT over LITH was observed at 100 mW/cm, and increasing the PS dose enhanced the cure rate, which can be attributed to PDT contribution. The interpretation of the results with 150 mW/cm (12.6–367 mW/cm^2^) is complicated by significant treatment-related mortality of 40 and 30% in mice treated with LITH alone and I-PDT, respectively (Fig. 4a). We observed elevated core temperatures in some of the mice that died. No such toxicity was observed in the rabbit model. Therefore, we speculate that mortality was due to expansion of heat in this small animal model.

Multiple studies have demonstrated that low light irradiance and high fluence will significantly improve local tumour control with EB-PDT in preclinical and clinical settings.^17,21,37^ Henderson et al. reported that 14 mW/cm^2^ and 128 J/cm^2^ could yield up to 80% cures, and an increase to 112 mW/cm^2^ with 128 J/cm^2^ resulted in no cures in mouse models.^17,37^ In contrast to EB-PDT, our results suggest that while exceeding the minimum light fluence, it is critical to deliver minimum intratumoural irradiance to 100% of the target tumour. In the mouse study with two fibers and 540 J/cm, there was a significant increase in cure rate by increasing intratumoural irradiance using 100 mW/cm in comparison to 60 mW/cm. However, very high intratumoural irradiance will impede the efficacy of PDT as seen in 400 mW/cm. High-temperature (*T* > 60 °C) thermal ablation is an established treatment for cancerous tumours.^39^ It is typically limited to the treatment of small tumours (<3 cm) not in the vicinity of major blood vessels (that act as heat sinks) and away from nerves. High-temperature thermal ablation can cause irreversible damage to nerves. In several small studies, I-PDT has been proven safe when applied to large locally advanced tumours next to facial nerves,^13,15,47,48^ offering a potential advantage over thermal ablation.

The FEM pretreatment planning was successfully used to translate the findings from the mouse studies to the NZW rabbit model, where the tumour size is close to human tumours. In previous publications, we demonstrated that FEM could be used to plan and guide interstitial light delivery in models mimicking human tumours.^35,36^ In this study, we used fibers, catheters, and CT imaging that are employed in the clinic. The impact of the Photofrin^®^-mediated PDT component, as compared to the thermal component, was much more pronounced in the rabbit than in the mouse model. Since the VX2 tumour is more akin to human tumours in both size and pathology, this finding is especially promising for the clinical use of I-PDT in locally advanced HNC.

We plan to follow this work with a multi-centre phase I/II clinical study to evaluate I-PDT with Photofrin^®^ in the treatment of patients with locally advanced HNC, utilising the guidelines in this manuscript. As a proof of concept, a treatment planning for a patient with locally advanced HNC who was amenable to receive I-PDT is shown in the supplementary material. The simulation was carried out with our FEM planning as previously described,^35^ and used in the present preclinical study. The treatment plan suggested that 12 cylindrical-diffuser fibers with 1, 2, and 3-cm long diffusers, delivering a light intensity of 200 mW/cm per fiber, would be required to deliver an intratumoural irradiance of ≥29.4 mW/cm² and a fluence of ≥45.9 J/cm² to 100% of the tumour.

## Electronic supplementary material

Supplementary material

## Availability of data and materials

The materials may be available through a request to Roswell Park Comprehensive Cancer Center. Some restriction may apply.

## References

[1] Zafereo ME (2009). The role of salvage surgery in patients with recurrent squamous cell carcinoma of the oropharynx. Cancer.

[2] Vermorken JB (2008). Platinum-based chemotherapy plus cetuximab in head and neck cancer. N. Engl. J. Med..

[3] Mendenhall WM, Mendenhall CM, Malyapa RS, Palta JR, Mendenhall NP (2008). Re-irradiation of head and neck carcinoma. Am. J. Clin. Oncol..

[4] Wong SJ, Spencer S (2008). Reirradiation and concurrent chemotherapy after salvage surgery: pay now or pay later. J. Clin. Oncol..

[5] McDonald MW (2011). ACR appropriateness criteria retreatment of recurrent head and neck cancer after prior definitive radiation expert panel on radiation oncology-head and neck cancer. Int. J. Radiat. Oncol. Biol. Phys..

[6] Janot F (2008). Randomized trial of postoperative reirradiation combined with chemotherapy after salvage surgery compared with salvage surgery alone in head and neck carcinoma. J. Clin. Oncol..

[7] Licitra L, Vermorken JB (2004). Is there still a role for neoadjuvant chemotherapy in head and neck cancer?. Ann. Oncol..

[8] Khuri FR, Shin DM, Glisson BS, Lippman SM, Hong WK (2000). Treatment of patients with recurrent or metastatic squamous cell carcinoma of the head and neck: current status and future directions. Semin. Oncol..

[9] Bauml J (2017). Pembrolizumab for platinum- and cetuximab-refractory head and neck cancer: results from a single-arm, phase II study. J. Clin. Oncol..

[10] Ferris RL (2016). Nivolumab for recurrent squamous-cell carcinoma of the head and neck. N. Engl. J. Med..

[11] Shafirstein Gal, Bellnier David, Oakley Emily, Hamilton Sasheen, Potasek Mary, Beeson Karl, Parilov Evgueni (2017). Interstitial Photodynamic Therapy—A Focused Review. Cancers.

[12] Agostinis P (2011). Photodynamic therapy of cancer: an update. CA Cancer J. Clin..

[13] Lou PJ (2004). Interstitial photodynamic therapy as salvage treatment for recurrent head and neck cancer. Br. J. Cancer.

[14] Jager HR, Taylor MN, Theodossy T, Hopper C (2005). MR imaging-guided interstitial photodynamic laser therapy for advanced head and neck tumours. AJNR Am. J. Neuroradiol..

[15] Jerjes W (2011). Photodynamic therapy: the minimally invasive surgical intervention for advanced and/or recurrent tongue base carcinoma. Lasers Surg. Med..

[16] Pinnacle Biologics, Inc. http://www.photofrin.com/wp-content/uploads/2013/02/prescribing-info.pdf (2013).

[17] Henderson BW, Busch TM, Snyder JW (2006). Fluence rate as a modulator of PDT mechanisms. Lasers Surg. Med..

[18] Coutier S, Bezdetnaya LN, Foster TH, Parache RM, Guillemin F (2002). Effect of irradiation fluence rate on the efficacy of photodynamic therapy and tumour oxygenation in meta-tetra (hydroxyphenyl) chlorin (mTHPC)-sensitized HT29 xenografts in nude mice. Radiat. Res..

[19] Busch TM (2002). Photodynamic therapy creates fluence rate-dependent gradients in the intratumoural spatial distribution of oxygen. Cancer Res..

[20] Sitnik TM, Henderson BW (1998). The effect of fluence rate on tumour and normal tissue responses to photodynamic therapy. Photochem. Photobiol..

[21] Henderson BW (2000). Photofrin photodynamic therapy can significantly deplete or preserve oxygenation in human basal cell carcinomas during treatment, depending on fluence rate. Cancer Res..

[22] Swartling J (2016). Online dosimetry for temoporfin-mediated interstitial photodynamic therapy using the canine prostate as model. J. Biomed. Opt..

[23] Yu G (2006). Real-time in situ monitoring of human prostate photodynamic therapy with diffuse light. Photochem. Photobiol..

[24] Zhu TC, Finlay JC (2006). Prostate PDT dosimetry. Photo. Photodyn. Ther..

[25] Davidson SR (2009). Treatment planning and dose analysis for interstitial photodynamic therapy of prostate cancer. Phys. Med. Biol..

[26] Kinsey JH, Cortese DA, Neel HB (1983). Thermal considerations in murine tumour killing using hematoporphyrin derivative phototherapy. Cancer Res..

[27] Seshadri M (2008). Light delivery over extended time periods enhances the effectiveness of photodynamic therapy. Clin. Cancer Res..

[28] Khurana D (2001). Characterization of a spontaneously arising murine squamous cell carcinoma (SCC VII) as a prerequisite for head and neck cancer immunotherapy. Head Neck.

[29] Suit HD, Sedlacek RS, Silver G, Dosoretz D (1985). Pentobarbital anesthesia and the response of tumour and normal tissue in the C3Hf/sed mouse to radiation. Radiat. Res..

[30] Lew YS, Kolozsvary A, Brown SL, Kim JH (2002). Synergistic interaction with arsenic trioxide and fractionated radiation in locally advanced murine tumour. Cancer Res..

[31] Shafirstein G (2009). Conductive interstitial thermal therapy (CITT) inhibits recurrence and metastasis in rabbit VX2 carcinoma model. Int. J. Hyperth..

[32] Parvinian A, Casadaban LC, Gaba RC (2014). Development, growth, propagation, and angiographic utilization of the rabbit VX2 model of liver cancer: a pictorial primer and “how to” guide. Diagn. Interv. Radiol..

[33] Rich LJ, Sexton S, Curtin L, Seshadri M (2017). Spatiotemporal optoacoustic mapping of tumour hemodynamics in a clinically relevant orthotopic rabbit model of head and neck cancer. Transl. Oncol..

[34] Bellnier DA (2003). Population pharmacokinetics of the photodynamic therapy agent 2-[1-hexyloxyethyl]-2-devinyl pyropheophorbide-a in cancer patients. Cancer Res..

[35] Oakley E (2015). A new finite element approach for near real-time simulation of light propagation in locally advanced head and neck tumours. Lasers Surg. Med..

[36] Oakley E (2017). Surface markers for guiding cylindrical diffuser fiber insertion in interstitial photodynamic therapy of head and neck cancer. Lasers Surg. Med..

[37] Henderson BW (2004). Choice of oxygen-conserving treatment regimen determines the inflammatory response and outcome of photodynamic therapy of tumours. Cancer Res..

[38] Shafirstein G (2016). Photodynamic therapy of non-small cell lung cancer. narrative review and future directions. Ann. Am. Thorac. Soc..

[39] Chu KF, Dupuy DE (2014). Thermal ablation of tumours: biological mechanisms and advances in therapy. Nat. Rev. Cancer.

[40] Gill PE, Murray W (1978). Algorithms for the solution of the nonlinear least-squares problem. SIAM J. Numer. Anal..

[41] Busch TM, Hahn SM, Evans SM, Koch CJ (2000). Depletion of tumour oxygenation during photodynamic therapy: detection by the hypoxia marker EF3 [2-(2-nitroimidazol-1[H]-yl)-*N*-(3,3,3-trifluoropropyl)acetamide]. Cancer Res..

[42] Sabino CP (2016). The optical properties of mouse skin in the visible and near infrared spectral regions. J. Photochem. Photobiol. B.

[43] van Gemert, M. J. C., Welch, A. J., Pickering, J. W. & Tan, O. T. in *Laser Treatment of Port Wine Stains* 789–829 (eds Welch, A. J. & van Gemert, M. J. C.) (Plenum Press, New York, 1995).

[44] Ritz JP (2001). Optical properties of native and coagulated porcine liver tissue between 400 and 2400 nm. Lasers Surg. Med..

[45] Henderson BW, Waldow SM, Potter WR, Dougherty TJ (1985). Interaction of photodynamic therapy and hyperthermia: tumour response and cell survival studies after treatment of mice in vivo. Cancer Res..

[46] Sapareto SA, Dewey WC (1984). Thermal dose determination in cancer therapy. Int. J. Radiat. Oncol. Biol. Phys..

[47] Karakullukcu B (2012). mTHPC mediated interstitial photodynamic therapy of recurrent nonmetastatic base of tongue cancers: development of a new method. Head Neck.

[48] Mimikos C, Shafirstein G, Arshad H (2016). Current state and future of photodynamic therapy for the treatment of head and neck squamous cell carcinoma. World J. Otorhinolaryngol. Head Neck Surg..

