# Supplementary material for: Irradiance controls photodynamic efficacy and tissue heating in experimental tumours: implication for interstitial PDT of locally advanced cancer
Source: Br J Cancer. 2018 Oct 24;119(10):1191–9. doi: 10.1038/s41416-018-0210-y (PMC6251027; doi:10.1038/s41416-018-0210-y)
Supplement: Supplementary file 1 — Supplementary material [file 41416_2018_210_MOESM1_ESM.docx]

**Supplementary material**: An example of treatment planning for interstitial photodynamic therapy of a locally advanced head and neck cancer. The plan suggests that 12 cylindrical diffuser fibers with 200 mW/cm in each fiber will be required to deliver an intratumoral irradiance ≥29.4 mW/cm², and a light dose of ≥ 45.9 J/cm² to 100% of the tumor, with a treatment time of 26 min.


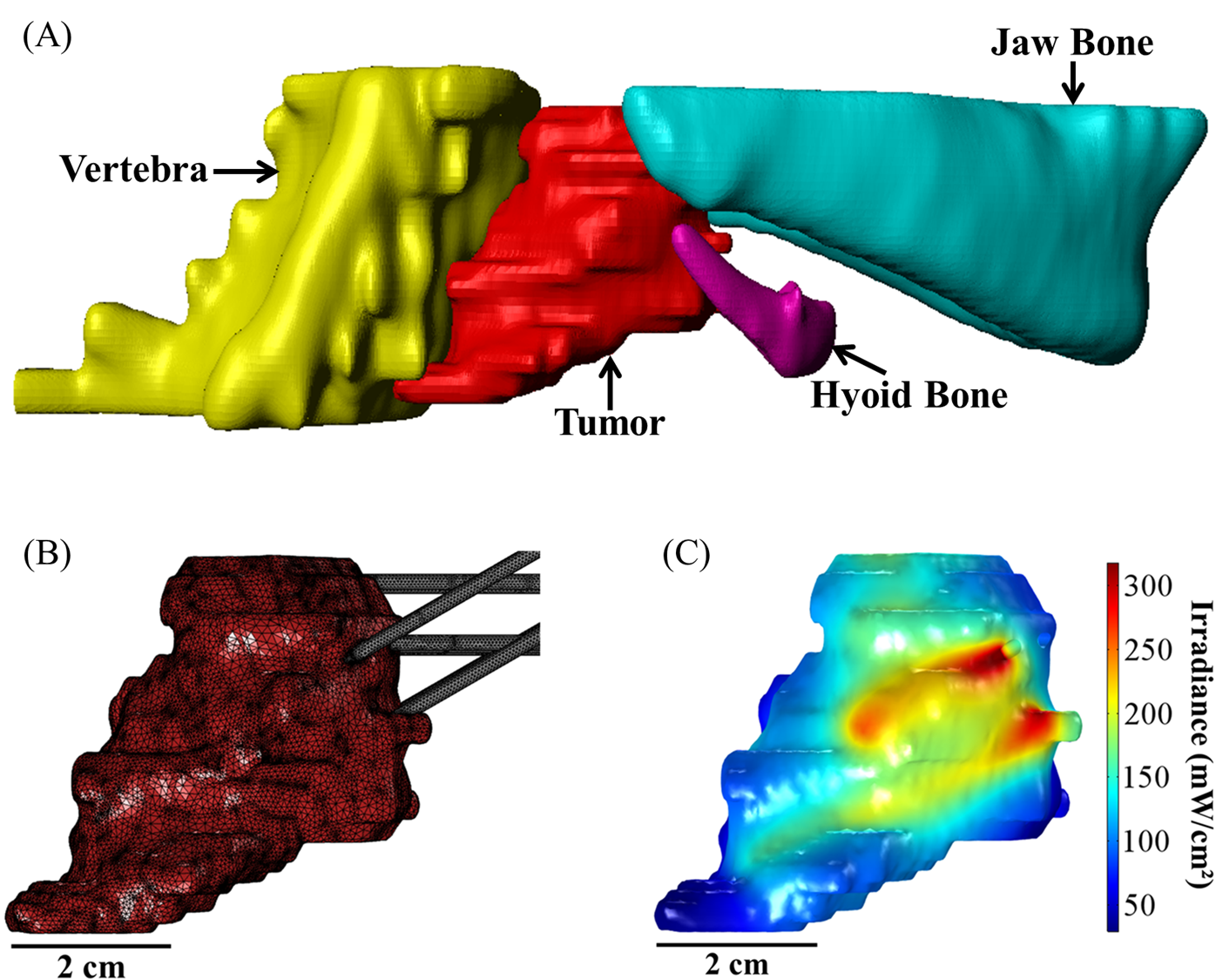


Finite element modeling treatment planning that can be used in clinical settings. (A) Segmentation from CT scans shows a 45 cm^3^ tumor (red), vertebra (yellow), hyoid bone (pink), and jawbone (blue). (B) A 3-dimensional mesh of tumor geometry, and fibers as black cylinders. (C) The light fluence rate distribution throughout the tumor. The optical properties used in this simulation were taken from Oakley *et al*. 2015, using absorption and scattering coefficients of 20 1/m and 2777.8 1/m, respectively, and anisotropy factor g=0.82.
